# Population Data BC: Supporting population data science in British Columbia

**DOI:** 10.23889/ijpds.v4i2.1133

**Published:** 2020-03-26

**Authors:** TK Ark, S Kesselring, B Hills, KM McGrail

**Affiliations:** 1 Population Data BC, School of Population and Public Health, UBC, Vancouver, BC, V6T 1Z3; 2 Centre for Health Services and Policy Research, School of Population and Public Health, UBC, Vancouver BC, V6T 1Z3

## Abstract

**Background:**

Population Data BC (PopData) was established as a multi-university data and education resource to support training and education, data linkage, and access to individual level, de-identified data for research in a wide variety of areas including human and community development and well-being.

**Approach:**

A combination of deterministic and probabilistic linkage is conducted based on the quality and availability of identifiers for data linkage. PopData utilizes a harmonized data request and approval process for data stewards and researchers to increase efficiency and ease of access to linked data. Researchers access linked data through a secure research environment (SRE) that is equipped with a wide variety of tools for analysis. The SRE also allows for ongoing management and control of data. PopData continues to expand its data holdings and to evolve its services as well as governance and data access process.

**Discussion:**

PopData has provided efficient and cost-effective access to linked data sets for research. After two decades of learning, future planned developments for the organization include, but are not limited to, policies to facilitate programs of research, access to reusable datasets, evaluation and use of new data linkage techniques such as privacy preserving record linkage (PPRL).

**Conclusion:**

PopData continues to maintain and grow the number and type of data holdings available for research. Its existing models support a number of large-scale research projects and demonstrate the benefits of having a third-party data linkage and provisioning center for research purposes. Building further connections with existing data holders and governing bodies will be important to ensure ongoing access to data and changes in policy exist to facilitate access for researchers

## Background/Introduction

Population Data BC (PopData) is a multi-university data and education resource that supports data linkage and access to individual-level, de-identified data for research on the determinants of human health, well-being and development. It was established in 2009, but has origins in the late 1980s with the creation of the BC Linked Health Data set (BCLHD), which pioneered the data linkage processes, access policy and process procedures in partnership with the British Columbia (BC) Ministry of Health (MoH).

PopData supports access to individual level, de-identified^1^ longitudinal data on the residents of BC across a large collection of health service, population health and other data sets. These are primarily administrative data, but there are a number of survey data sets and continued expansion to other types of data, including contextual data that describe neighbourhoods, communities and environmental exposures. PopData strives to deliver new services, data sets and supports in response to evolving needs and aspirations of the research community.

PopData is physically located at the University of British Columbia (UBC) in the School of Population and Public Health. It operates under the guidance of an advisory board that includes representatives from all research-intensive Universities in the province as well as researchers and data stewards^2^. PopData receives operational funding from its data provider partners (e.g. BC Cancer, BC MoH, Human Early Learning Partnership, and WorkSafeBC), other partners (e.g. Michael Smith Foundation for Health Research (MSFHR)) and cost-recovery charges to researchers. It receives strategic funding for new services and innovation from partners (e.g. the BC SUPPORT Unit, the provincial Data Innovation Program) and competitive grants (e.g. MSFHR and the Canadian Institutes for Health Research).

## Approach

### Population Setting

British Columbia is Canada’s western-most province, bordered on the west by the Pacific Ocean, the east by the Rocky Mountains and the province of Alberta, and the north by the Yukon Territory. Its population in 2018 was just under five million in a land mass of a little under one million square kilometers (four times the size of the UK, or just a little smaller than France and Germany combined). More than half of the population is concentrated in the southwest corner of the province.

In 2018, BC’s population was aging slightly faster than the Canadian average, with 18.3% of the total aged 65+ [1]. Based on the 2016 census, 28.2% of BC residents were immigrants who arrived from a wide range of countries with the dominant regions being Asia (61.0% of the total immigrant population) and Europe (24.7%) [2]. The average life expectancy is 82.5 years, and BC’s population has lower rates of obesity (21.3% vs. 26.3%), smoking (14.1% vs. 17.4%) and higher rates of physical activity (65.7% vs. 57.7%) than the Canadian average [3].

The delivery of health and social care is largely the responsibility of provincial and territorial governments under a federal framework that stipulates universal, first-dollar for “medically necessary” services, meaning services provided by physicians and/or in acute care settings [4]. Other public coverage, for example for prescribed drugs, residential care, and other community-based services, is offered at the discretion of individual jurisdictions. British Columbia has a public coverage plan for pharmaceuticals that requires out-of-pocket payments that vary with year of birth, and for long-term care and community-based services that depend on both need and income [5, 6]. Private insurance is offered by some employers, and is limited to services that are not covered under the public plan [7]. Individual coverage outside employer-based plans is very rare. Social services are a mix of universal (e.g. education) and target (e.g. income assistance) programs.

### Operating model

In the late-1980s through to the mid-1990s, the BCLHD brought together data from BC health care services, BC Cancer and WorkSafeBC, and made these data available for researcher requests starting in 1996. The motivation for those early developments was to ensure long-term archiving and availability of historical administrative data and to invest in building data infrastructure that could serve many research needs. There was no formal or coordinated data access process prior to the start of this service. The timing of this initial service coincided with the proclamation of the BC *Freedom of Information and Protection of Privacy Act* (FIPPA), which includes allowances for research use of personal health information.

In 2005, academic partners in the BCLHD were awarded a large (almost $8m CAD) grant from the Canada Foundation for Innovation to further extend infrastructure. This grant was used to renovate space for secure housing of servers within a layered secure physical setting, and to further develop data linkage capacity and data sources. Further funding from the MSFHR in 2007 and 2012 supported refinement of service processes, and the creation of an education and training unit. The whole of the operation was rebranded in 2008 as Population Data BC, or PopData.

One guiding motivation for PopData is to make continuous improvements in the efficiency and transparency of the data access process for researchers and data providers, and to ensure transparency to the public. The policies that are implemented by PopData are created in consultation with a Data Stewards Working Group (DSWG), which is coordinated by PopData, and includes individuals from data providing organizations. PopData consulted with this group to draft and then implement a policy framework for access to linked and unlinked datasets, communications initiatives to ensure researchers understood the process, and privacy and compliance requirements for accessing and using the data.

PopData continues to work with the DSWG to make process improvements, and to introduce service innovations that respond to evolving needs given changes in technology, researcher ambitions, and data steward expectations. For example, PopData proposed to the DSWG the development of technical and procedural policies for a secure research environment (SRE); this new (and at the time quite unique) service was launched in 2010. The SRE allows researchers remote access to individual level-data that are retained in one central location and enables ongoing oversight and management of access controls by PopData. For example, this includes suspension of access if ethics has expired, logging of all transfers of analytic outputs and seamless data updates as required.

PopData houses data on site, and in some cases has arrangements with data stewards to pull data from a central data store; these arrangements are determined based on each data steward’s needs and preferences. PopData is a trusted third party for data linkage. It does not have a research mandate, and uses and/or discloses data only under documented authority from data providers.

PopData continues to expand the number of datasets available for research beyond the health domain, and now provides secure storage and access for other organizations. It is continually evaluating data linkage processes in collaboration with international partners, and adapting new practices where warranted.

### Architecture and information technology

PopData has established a high level of physical, technical, and organizational security for all data in its custody, meeting or exceeding well-recognized International Organization for Standardization (ISO) requirements for information security (more detail is provided in the Privacy by Design section below). In 2009 and 2014, external third-party consultants were engaged to conduct a systems and security review of PopData’s information security practices and confirmed its security strengths and safeguards against ISO requirements.

While PopData differentiates between data that include (or potentially include) personally identifying information (i.e. identifiers) and data that do not (i.e. content data), all data are considered to be highly sensitive and are protected with appropriate safeguards. Identifiable information is defined as that which can be used, linked, matched or manipulated by a reasonable method to identify subject’s identity, such as surname, given name, date of birth (year, month and day), address and identifying unique numbers (e.g., Personal Health Number). Content data refers to data that have been recorded, collected, observed, or generated for research or administrative purposes (e.g., ICD-9 codes). This information does not contain any direct personal identifiers.

The data received from Data Stewards are stored, accessed and processed by a limited number of PopData analysts in a physically secure location on a separate internal network. When PopData receives a new data file, limited data validation is conducted and identifiers used for linkage are separated from content data. At this point, both data sets have a common PopID (a generated number unique to each record) applied to them so that the ID resulting from data linkage can be attached to the content data. This ID is then replaced with a project specific ID when data are provisioned to an approved research project.

Sensitive data storage and data linkage are conducted on servers that are separate from those used to store content data and to create research extracts for approved research projects. Servers holding sensitive data are part of a network area that has no connection to the internet. All PopData servers are located on-site, in a separate, secure, climate-controlled room to which a limited number of specialize staff have access. 

### Governance, legislation and management

PopData developed and implemented policies and procedures reflecting BC legislative requirements concerning collection, use, and disclosure of *Personal Information found in the BC Freedom of Information and Protection of Privacy Act (FIPPA)* and the BC *E-Health (Personal Health Information Access and Protection of Privacy) Act*^3^.

Individual Information Sharing Agreements (ISAs) exist between each data provider and PopData to permit storage, management and provisioning of data for approved research projects, and use of specific variables for data linkage. Specific agreements are in place between providers and PopData to maintain and append the Population Directory (the primary data linkage file), and use it for data linkage of approved projects. This is different from the agreements in place for approved research projects – a research agreement exists between the data providers and the researchers.

PopData operates within a governance and management framework that includes the DSWG and an advisory board. The DSWG includes Data Stewards from each organization whose data access is facilitated by PopData. While PopData facilitates data access for research purposes, data ownership and stewardship (governing rules around the use and approval of access to the data) are retained by the Data Stewards who partner with PopData.

### Consent Model

The obligation for obtaining consent(s) rests with those public bodies (i.e. Data Stewards) and researchers who originally collect the data. PopData does not perform any primary collection of personal information and is only engaged in secondary use or secondary disclosure of personal information initially collected by other public bodies or individuals.

Pursuant to section 33 of FIPPA, the designated public bodies are permitted to disclose personal information to PopData. Pursuant to Section 35 of FIPPA, PopData and researchers with approved access to data collected by the designated public bodies are not required to seek individual consent for the use of those data for research and statistical purposes.

PopData relies on public bodies to collect personal information in a lawful manner and in accordance with the requirements of FIPPA. Where consent is required, PopData relies on the public bodies overseeing the initial collection of personal information to have obtained the appropriate consent(s) required for collecting and using the personal information. Research ethics board and Data Steward reviews confirm whether the consent(s) is/are appropriate for the requested uses of the data, and this is part of the approval process. FIPPA governs PHNs and identifying information, but does not prescribe what constitutes the “identifier” vs. “content” data described above. 

### Privacy by design

PopData’s privacy mandate is to meet and exceed all relevant legal, ethical and legislative guidelines for data protection and privacy [8]. The tenets of individual privacy and data protection are embedded in all aspects of PopData operations, and include physical, technical and procedural measures. PopData is built on a set of core privacy principles that include privacy by design. This includes preventative and proactive safeguards, embedding privacy into design and as a default, full lifecycle protection of data, and independent verification.

PopData has physical controls and technical security that consists of three zones: purple, red and yellow. The purple zone houses the data servers, with fortified walls and a separate alarm. This zone and the servers are accessible to a limited number of employees. The red zone is a separate locked room with video surveillance, fortified walls and alarms. A restricted number of data analysts access the encrypted raw and identifiable data stored in the purple zone using two-factor authentication through a network moated in the physical red zone. The yellow zone is an added extra layer of security and is a locked zone to which every PopData employee has access. Computers in the yellow zone can communicate externally, but are protected by firewalls, and do not contain any identifiable or raw data.

Other important privacy by design principles include: a) separation of identifiers from research content, b) the creation of the SRE so individual level data are located in a secure, central server, and not an individual’s computer, c) encryption of data throughout the life cycle (transfer, back-ups, destruction) of a project, and d) auditing, which includes documentation of who accessed or transferred and activities on/with data. These principles follow the international standards (such as ISO), but are customized for facilitating research in Canada.

### Data linkage

PopData’s data linkage approach is aligned with provincial and federal privacy-related legislative policies, described in the governance, legislation and management section, and interpretation of these policies by its data provider partners. PopData has agreements in place to conduct data linkage as data arrive from data stewards, which at present is mostly annually. This process of “linking once” allows the provisioning of data in a timely and efficient manner. PopData also has a process for “one-off” linkage, for example in cases where researchers have an external data set they wish to link to other data. In this case, direct identifiers are brought together and used only after there is approval in place from all relevant data providers.

The “Population Directory” is the core linkage file maintained by PopData and serves as a spine for data linkage. It includes all the individuals about whom PopData has information. It contains personal information such as name, address, date of birth, and other relevant identifying information, as well as a consistent, encrypted identifier (a “Linkage ID”) that uniquely identifies each individual. This Population Directory has been built with Medical Services Plan (MSP) Registration & Premium Billing (R&PB) data going back to 1985 and is updated upon receipt of each new R&PB file. MSP is BC’s public health insurance plan, and when each BC resident enrolls (which is required for nearly all residents), they are issued a (nearly) unique lifetime identifier for health care called a Personal Health Number (PHN). As a result, this dataset provides the largest representative coverage of residents in BC to support high quality data linkage, including PHN, first, middle and last name, date of birth, sex, and postal code. The Population Directory captures all changes (e.g. a postal code history) and name permutations (such as maiden name/married name). The Population Directory covers most of the BC population with the exception of populations that are the responsibility of federal coverage, such as federal prisoners and employees of the Royal Canadian Mounted Police.

PopData conducts a combination of deterministic and probabilistic linkage and clerical review for competing matches. This is currently undertaken using software developed by PopData staff largely following the data linkage theories set forth by Newcombe (1988)[9] and operationalized by Fellegi and Sunter (1969)[10], and Jaro and Winkler [11, 12]. For core holdings, records are linked and stored with the PopDataID to facilitate provisioning of data in a timely manner.

Linkage weights and a data linkage outcome string are derived to describe how a record pair was a match and assess the quality of potential links. The outcome string represents the field comparisons used for data linkage. For instance, if nine variables are used as identifiers for data linkage, this will result in a nine-digit numeric string, with each column relating to one of the nine variables used in the linkage process (e.g., Personal Health Number (PHN), surname, given name, middle name, sex, date of birth [year, month, date of day], and postal code). An outcome string of 111211110, represents a record pair that has exact agreement on PHN, given and surname, gender, all aspects of date of birth (year, month and day). However, there is partial agreement on middle name, and the postal code is missing^4^. Comparison outcome component weights are applied based on match and non-match probability estimates and aggregated to a record weight. The record weight, outcome string and clerical review is used to establish rules that facilitate and identify accepted matches.

Once data linkage is complete, the linkage ID is converted to a PopDataID, and this ID is attached to the research content using the unique record ID. When data are provisioned for a research project, the PopDataID is replaced using a research project specific key to ensure researchers cannot identify individuals across different projects. The retention of the PopDataID on the research collections allows for an efficient and systematic approach to provisioning data for approved research projects.

### Data linkage keys

The variables used to conduct data linkage vary by dataset, and the variables are brought together for the purpose of data linkage only. When conducting linkage, only the data linkage variables (unique and demographic identifiers) are used. The linkage keys vary as they are limited to what is available in the target data set; this inevitably impacts the linkage rates. In some cases, additional identifier variables within specific domains are used to complement identifiers from MSP for a specific linkage, e.g. mother’s PHN to assist with linkage of newborns. Access to data linkage keys is limited and only available to those conducting data linkage at PopData.

### Data sources

PopData currently manages data from various federal and provincial government Ministries and agencies and research groups. Many of these data sets are population-based, meaning they cover all relevant individuals in the province. These data sets are linkable to each other and other external sources, including data collected by researchers, pending approval by Data Stewards on a case-by-case basis. The amount of data available related to each individual in BC varies based on their life experience and interactions with various health and social systems for which PopData holds data. Table 1 provides a list of the collections managed by PopData for research use.

**Table 1: Measures and characteristics available in the pseudonymised Cafcass data extract.** *historical data is available for select fields that date back as far as 1916. **data do not represent an individual, they are defined at geographic postal code level in British Columbia

Areas	Available datasets (and Data Steward)	Coverage start date	Volume (as of 2019)	Updates
Health Care and Health Services	Medical Service Plan Payment Information (BC Ministry of Health)	1985	2.5 billion	Annually
	PharmaCare (BC Ministry of Health)	1986	620 million	Now part of PNET
	Discharge Abstract Database (Hospital Separations) (BC Ministry of Health)	1985	22.7 million	Annually
	Home And Community Care (Continuing Care) (BC Ministry of Health)	1990	19.6 million	Annually
	Mental Health (BC Ministry of Health)	1986	11 million	Last updated in 2011
	BC Cancer Registry (BC Cancer)	1985	672 thousand	Annually
	Patient Centered Measurement (BC Ministry of Health), various surveys	Varies by survey	30,000	NA
	Perinatal Data Registry (Perinatal Services BC)	2000	898 thousand	Annually
	PharmaNet (BC Ministry of Health)	1995	55 million	Project specific basis
	Pharmacare (BC Ministry of Health)	1985	660 thousand	NA
	National Ambulatory Care Reporting System (NACRS) (BC Ministry of Health)	2011	7.2 million	Project specific basis
Population and Vital Statistics	Consolidation File (MSP	amp; Premium Billings) (BC Ministry of Health)	1986	794 million	Quarterly
	Vital Statistics Births (BC Vital Statistics Agency)	1985	2.7 million	Annually
	Vital Statistics Deaths (BC Vital Statistics Agency)	1985	873 thousand	Annually
	Vital Statistics Marriages (BC Vital Statistics Agency)	1985	706 thousand	Annually
	Vital Statistics Still Births (BC Vital Statistics Agency)	1985	2.5 million	Annually
Demographics and Life Course	BC Generations Project (BC Cancer)	2009	29 thousand	Annually
	Permanent Residents (Immigration, Refugees and Citizenship Canada)	1985	1 million	Annually
	Income Band (Statistics Canada)	1992, 2002, 2006	599 thousand**	Intermittent
Occupational	WorkSafeBC Claims (WorkSafeBC)	1981*	108 million	Annually
	WorkSafeBC Firm Level Files (WorkSafeBC)
Childhood	Early Development Instrument (EDI) (Human Early Learning Partnership)	1999	258 thousand	Annually
Education	Ministry of Education (MED)	1991	2.6 million	Annually
Environment	Canadian Urban Environmental Health Consortium (CANUE)	1983	128 thousand**	Annually

Frequency of data updates vary by data set. One of PopData’s goals is to provide data that are up-to-date rather than on an annual basis. Once data are available to PopData, it takes a month to validate and link before they are available for research requests and use.

### Data access

Requirements for data access are governed by The Research Data Access Framework (RDAF)^5^, a consensus-based document developed and approved by PopData and all Data Steward partners that conforms with FIPPA. Details on the RDAF and researcher eligibility criteria can be found in figure 1.

Figure 1: The Research Data Access Framework The RDAF outlines the criteria which a request for data must meet in order for it to be considered eligible:

- Be for the time-limited purpose of addressing a specific set of research questions,
- Not involve use of data for administrative or any other non-research purpose, or for ongoing programs of research, unless specifically approved,
- Be in the public interest, for example, improves the welfare of the population,
- Not be proprietary research such as research done for commercial marketing purposes,
- Have scientific merit,
- Have approval from a recognized Research Ethics Board, as defined by the *Tri-Council Policy Statement: Ethical Conduct for Research Involving Humans*^6^.

In addition to the requirements for research projects listed in the RDAF, there are criteria that the researcher must meet in order to be eligible for data access:

- the researcher is either a student, teacher, or other individual enrolled, appointed or employed by a BC university, college or provincial institute as defined under relevant BC legislation^7^ or another equivalent educational institution in another jurisdiction outside B.C. but within Canada, or
- any other individual agreed to by the relevant Data Steward of the Public Body.

Only researchers who will conduct their analyses in Canada are eligible.

Researchers apply for access to PopData-held administrative data using the Data Access Request (DAR) Online^TM^ form. This form captures relevant information regarding a researcher’s request for Data Stewards to assess whether the project meets approval criteria. The sections in the form include: funding, external peer review, ethical review, project description, study population, data required, privacy impact assessment, and data storage.

Members of the Data Access Unit (DAU) at PopData coordinate and facilitate the various stages of the data access process for researchers from initial enquiries, DAR planning, completion and submission to Data Steward(s). When the researcher’s DAR is submitted, the DAU undertakes an initial review for completeness prior to submitting the DAR to the relevant Data Stewards for their full review and approval. This minimizes the potential back-and-forth that would otherwise transpire, especially for researchers who have never submitted a DAR. The DAU communicates the decision made by the Data Stewards regarding project approval to the researchers.

Following approval by the relevant Data Stewards, the researcher must complete privacy training and sign a number of forms before data can be provisioned (see Figure 2).

Figure 2: Required Process and Forms for Data Provisioning

- Researcher Agreement with the relevant Data Stewards for their request (this provides a legally enforceable framework for data sharing between the Data Steward and the researcher)
- Confidentiality pledge
- Services Agreement with PopData (lays out the services PopData will provide and the expectations from the researcher)
- Cost quote prepared by PopData (signed by the researcher in acceptance of the cost)
- Researcher account created with PopData and researcher set up for SRE access

Data extracts are created based on the approved cohort definition in the data access application request. PopData works with the researchers to ensure the operationalization of the cohort is correct and aligned with their approved request. Approved data are provisioned and released to the SRE and accessible only by the approved researchers on the project. Generally, researchers access their research extracts using PopData’s SRE, however exceptions may be agreed upon by the Data Stewards involved for data to be released to external safe havens. The SRE is accessed virtually (currently from anywhere in Canada) using two-factor authentication. Each user must have their own credentials, and access is enabled to separate project-specific extracts. The SRE includes a comprehensive set of software for analysis (e.g. SAS, Stata, R, Python – see here for a full list^8^), and ensures safe storage and back of data and analyses.

Users of PopData’s SRE currently use a self-vetting system for removal of analytic output from the SRE, following specific guidelines outlined in their research agreements. PopData’s privacy training and materials available on the PopData website outline what are considered safe and unsafe outputs from the SRE, e.g. no record-level data are allowed. All outputs submitted for export trigger a reminder warning to the user to ensure that the export is allowed. Once confirmed, export requests are audited, and are automatically scanned for type, size and content. Suspicious files are blocked for manual review, which are conducted by PopData staff. Successful self-vetted outputs are checked at random. Copies of all file transfers (import, export and blocked transfers) are archived at PopData. The removal of research outputs from the SRE uses a “yellow folder” system that was built by PopData. There is a formal incident / breach reporting system that is triggered any time an issue is identified, such as an attempt to remove a disallowed file. In this case, all access for the specific project is temporarily suspended, the incident is reported to relevant data providers, and a follow-up investigation is conducted with the researcher(s). Appropriate corrective action is taken, such as additional privacy training, and a formal write-up closes the file. There have been no breaches of potentially identifying information at any time since 1996.

The average elapsed time from request submission to data access is four to six months, depending on the complexity of the request. Some of the factors that impact this timeline include: cohort definition, number of variables that require additional justification, number of data collections requested (and thus the number of data stewards involved in review and approval), and whether the data requested are already part of the PopData holdings. These timelines are monitored closely and are the target of ongoing policy attention, with the intent of instituting new processes that will enable some requests (that meet pre-defined criteria) to be met within a matter of weeks rather than months.

PopData’s primary role is to maintain, link and provision data for approved research projects and not to participate in or provide any form of analytical support for research projects. PopData does, however, provide a number of resources to support population level research with a particular focus on health related data, such as Snippets, a resource to document and share analytic code, as well as courses, webinars, training certifications, and workshops.

### Noteworthy outputs

Population health research focuses on populations/sub-populations and conducts studies that seek to describe and explore the relationship between health outcomes and the determinants of health (e.g., socio-economic status, education, etc.) to improve population health. Researchers who have access to linked data through PopData have contributed to the growing body of population health research and their studies have led to evidence-based policy-making decisions to improve population health and address disparities. Examples of these studies are as follows:

 1. *Does where you live affect your health?*

The Border Air Quality study is a multi-institutional study that investigated the impact of exposure to a range of air and its impact on health outcomes in the Georgia Basin-Puget Airshed [4]. This study examined data from over 80, 000 mothers and children, and over 500,000 adults. Data for this study included Medical Service Plan (MSP), Hospital Separations (DAD), Perinatal, Vital Statistics Births and Deaths, Census data, data from the BC Ministry of Environment, the researcher’s own collected data, and data from metro Vancouver municipalities. The results of this collaborative initiative found that mothers living within 50 meters of a highway were associated with a 26% increase in small gestational birth weight, 11% increase in low full-term birth weight, and 21% more likely to be born pre-term (less than 37 weeks) [13]. Six percent were more likely to develop infant bronchiolitis requiring medical attention [14, 15], 13% more likely to develop new childhood asthma [16] and 7% more likely to develop middle ear infections[17] from ambient air pollution. Further, Gan et al. [18, 19] found residential proximity to traffic may partly explain the association between road traffic and cardiovascular outcomes. The benefits of such a large-scale collaborative initiative and access to data allowed multiple factors to be examined by researchers with different expertise to work together on the same dataset.

 2. *Are there early-warning signs of multiple sclerosis?*

Wijnands et al. [20] examined the traits and symptoms exhibited by multiple sclerosis (MS) patients five years prior to their first recognized symptoms (referred to as prodrome) of MS from 1984 to 2014 across four Canadian provinces (BC, Saskatchewan, Manitoba and Nova Scotia) using administrative health datasets (PharmaNet, MSP Registration and Premium Billing, Consolidation Registry, Hospital Separation and Deaths). In the five-year period before the first MS symptom, patients with MS were three times more likely to experience fibromyalgia and two times more likely to have irritable bowel syndrome than patients without MS. In addition, patients with MS had higher rates of migraines and mood or anxiety disorders (e.g., depression, anxiety, bipolar) than non-MS patients. Given the higher rates of these conditions, these patients also had higher use of medications for musculoskeletal, nervous system and genito-urinary tract disorders along with antidepressants and antibiotics. This is one of the first comprehensive MS studies to use medical records in this capacity to study MS prodrome and identify the need for future research that examines differences across gender, age and ethnicity. This study provides valuable insight and awareness for practicing physicians of the early potential signs of MS and prompt patient management strategies early on in MS.

 3. *Are home births riskier than having a baby in the hospital?*

Janssen et al. [21] conducted a study to examine the effects of having a planned homebirth in the presence of a registered midwife to a hospital birth with a registered midwife or physician for women with low risk pregnancies. The data for this study were obtained from Perinatal Database Registry, Vital Statistics (Births and Deaths) and Midwifery data, Hospital Separations (DAD) and the Consolidation File. The study found planned home births with a registered midwife had fewer perinatal deaths and were less likely to have obstetric interventions or other outcomes (e.g., perineal tear, postpartum hemorrhage) than a planned hospital birth with a registered midwife or physician in low risk pregnancies. The results of this study had wide spread implications for the regulation of midwifery in Canada and provided valuable insight for women deciding between a hospital or home birth.

Further case studies and research outputs are available on PopData’s website: https://www.popdata.bc.ca/RIA

## Discussion

Providing efficient and cost-effective access to linked administrative data sets for research was a major driving force in establishing PopData and its predecessor. The value of these data is demonstrated through a variety of research projects accessing PopData holdings. There is ongoing work being done to ensure that governance, legislation and policy is kept current, including to ensure timely approvals, and to consider new developments such as interest in machine learning.

Based on two decades of learning, there are areas identified where further development and research is needed. These include, but are not limited to: a) evolving governance and policy models to facilitate programs of research, low-risk accessible and/or cleaned data sets with an expedited review, and cloud computing for computational demanding analyses; b) training, development and implementation of data management and analysis of large data files through parallel processing techniques for researchers, c) evaluating the use of new data linkage techniques such as privacy preserving record linkage (PPRL); d) on-going updates/maintenance of metadata to ensure the research outcomes accurately reflect the state of the data; e) further training and education opportunities; and f) engaging with the public around appropriate rules for access to and use of complex linked data.

## Conclusion

PopData has been conducting data linkage and facilitating data access for research purposes on a large scale for more than two decades. PopData continues to support access, linkage and provisioning of individual-level, de-identified research data that has led to over 350 research projects and over 1550 research outputs (e.g., publications, presentations and dissertations) to date. The sources and type of data available to researchers have grown over time to include not only administrative health data, but also survey and contextual data that can facilitate research in a wide variety of areas. PopData has created many systems to enable data access for researchers and strives to continually improve operational efficiency, such as by creating a harmonized, single-point of access for researchers to request data both as a process and technological tool (e.g., DAR online). This also includes the development and refinement of technological practices to ensure data is provisioned in a timely manner, while minimizing disclosures risks.

Much of the future development of PopData will involve improving governance, access to reusable datasets, and creating tools for researchers to manage large data sets and conduct analyses in a time-efficient manner. As the amount of data increases and researcher ambitions continue to evolve, there will be a need to explore policies and governance around housing data in the cloud, federating data sets, distributed analysis, and techniques such as privacy preserving record linkage. PopData’s involvement with the International Population Data Linkage Network and connections to other similar groups will be instrumental in assuring we continue to help establish and operate under best practices for population data centres.

### Ethics

Ethical approval was not required because this work did not involve research with human participants.

## Footnotes

1 No personal identity information (e.g., surname, given name or address) or unique identifiers (e.g., personal health numbers) are provided.

2 Data providers assign an individual in the organization with the role of data steward. The specific responsibilities of data stewards may vary from organization to organization, but all retain adjudication and approval responsibility over requests for access to data. PopData works with these individuals in evolving data access and approval processes.

3 Details on the relevant legislation and elements that impact PopData work can be found here: https://www.popdata.bc.ca/privacy/policies/legislativeframework and http://www.bclaws.ca/Recon/document/ID/freeside/96165_00

4 A value of 1 refers to agreement, 9 refers to disagreement, 0 is missing and values ranging between 2 to 8 quantifies the distance of partial agreement between strings.

6 See Chapter 6: Governance of Research Ethics Review in the Tri-Council Policy Statement: Ethical Conduct for Research Involving Humans, available here: http://www.pre.ethics.gc.ca/pdf/eng/tcps2/TCPS_2_FINAL_Web.pdf

7 Eligible institutions and the relevant BC legislation are detailed on this website: https://www.popdata.bc.ca/dataaccess/rdaf/eligibility

8
https://my.popdata.bc.ca/html/SRE/software.html
